# Analysis of the NLRP3 inflammasome components expression in triple-negative breast cancer patients with and without BRCA1 mutations

**DOI:** 10.1038/s41598-026-43392-0

**Published:** 2026-03-21

**Authors:** Sara Socorro Faria, Susan Costantini, Elena Di Gennaro, Vladmir C. Cordeiro de Lima, Victor Piana Andrade, Cristiano de Pádua, Vinícius Duval, Marcia Maria Chiquitelli Marques, Kelly Grace Magalhães

**Affiliations:** 1 Laboratory of Immunology and Inflammation, Department of Cell Biology, University of Brasilia, Brasilia, DF Brazil; 2 Experimental Pharmacology Unit, Istituto Nazionale Tumori - IRCCS - Fondazione G. Pascale, 80131 Naples, Italy; 3 Department of Medical Oncology, A.C. Camargo Cancer Center, Sao Paulo, SP Brazil; 4 Department of Anatomical Pathology, A.C. Camargo Cancer Center, Sao Paulo, SP Brazil; 5 Department of Medical Oncology, Barretos Cancer Hospital, Barretos, SP Brazil; 6 Molecular Oncology Research Center, Barretos Cancer Hospital, Barretos, SP Brazil

**Keywords:** Inflammasome, Mutation, Triple-negative breast cancer, Breast cancer, Tumour immunology

## Abstract

Triple-negative breast cancer (TNBC) is an aggressive and immunogenic breast cancer subtype frequently associated with BRCA1 alterations. Inflammation and innate immune sensing pathways, including inflammasomes, play complex and context-dependent roles in tumor progression and anti-tumor immunity. However, the prognostic significance of inflammasome components in TNBC remains poorly defined. We evaluated the protein expression of key inflammasome components including NLR family pyrin domain-containing 3 (NLRP3), PYD and CARD domain-containing protein (PYCARD), caspase-1 (CASP1), and interleukin-18 (IL-18) by immunohistochemistry in tumor samples from 88 TNBC patients stratified by BRCA1 status, including pathogenic germline mutations, promoter hypermethylation, and wild-type tumors. Survival analyses were performed using Kaplan–Meier estimates and Cox proportional hazards models. Lower CASP1 expression was significantly associated with smaller tumor size (*p* = 0.005), whereas lower NLRP3 expression was associated with axillary lymph node metastasis (*p* = 0.003). No significant association was observed between inflammasome protein expression and BRCA1 mutation or promoter hypermethylation status. Importantly, low NLRP3 expression was independently associated with worse disease-free survival (DFS) (hazard ratio (HR) = 3.15, 95% confidence interval (CI) = 1.36 to 7.30, *p* = 0.007) and overall survival (OS) (HR = 2.63, 95% CI = 1.19 to 5.79, *p* = 0.01). These findings indicate that reduced NLRP3 expression is associated with unfavorable prognosis in TNBC. Although exploratory in nature, this study highlights the potential relevance of inflammasome components as prognostic biomarkers in this aggressive breast cancer subtype and warrants further validation in independent cohorts.

**Supplementary Information:**

The online version contains supplementary material available at 10.1038/s41598-026-43392-0.

## Background

Triple-negative breast cancer (TNBC) accounts for approximately 10–20% of all breast cancer cases^1^. It is defined by the lack of expression of estrogen receptor (ER), progesterone receptor (PR), and human epidermal growth factor receptor 2 (HER2)^2^. This subtype is biologically heterogeneous and characterized by distinct molecular alterations, including specific somatic mutations and copy number variations^3^. Notably, approximately 15–20% of TNBC cases are associated with pathogenic germline variants in the BRCA1 gene^4,5^. The proteins encoded by BRCA1 and BRCA2 play essential roles in the maintenance of genomic stability through homologous recombination (HR)-mediated repair of DNA double-strand breaks^6,7^.

Among breast cancer subtypes, TNBC is considered one of the most immunogenic. This feature has been attributed to its high levels of tumor-infiltrating lymphocytes (TILs)^8^, increased clonal diversity, and frequent chromosomal alterations^9,10^. In particular, tumors from women harboring BRCA1 mutations exhibit enhanced expression of CD8 + T cell-related gene signatures and distinct copy number profiles^11^. These immunological and genomic features provide a biological rationale for the clinical application of immune checkpoint inhibitors (ICIs) in TNBC.

Within the tumor microenvironment (TME), inflammatory signaling plays a central role in shaping tumor progression and immune responses. One of the key mediators of innate immune activation in this context is the inflammasome, a multiprotein complex composed of a cytosolic pattern recognition receptor (PRR), the adaptor protein ASC (apoptosis-associated speck-like protein containing a CARD, also known as PYCARD), and the inflammatory protease caspase-1 (CASP1)^12^. Among the nucleotide-binding oligomerization domain-like receptor (NLR) family, NLRP3 (NOD-, LRR-, and pyrin domain-containing protein 3) is the most extensively studied inflammasome sensor. Previous studies have shown that NLRP3 activation can promote tumor progression by favoring the expansion of myeloid-derived suppressor cells (MDSCs)^13^ and by suppressing the cytotoxic activity of natural killer (NK) cells and CD8 + T lymphocytes^14^. However, accumulating evidence indicates that the functional consequences of NLRP3 signaling are highly context-dependent and may vary according to tumor type, genetic background, and microenvironmental cues.

Recent clinical studies have demonstrated enhanced anti-tumor immune responses in TNBC patients treated with ICIs, including antibodies targeting programmed death-1 (PD-1) and cytotoxic T-lymphocyte-associated protein 4 (CTLA-4)^15,16^. In murine orthotopic tumor models, anti-PD-1 therapy was shown to activate the NLRP3 inflammasome and promote myeloid cell recruitment, an effect that was dependent on CD8 + T cells^17,18^. In addition, in TNBC tumors with wild-type BRCA genes, the combination of low-dose DNA methyltransferase inhibitors (DNMTi) and poly(ADP-ribose) polymerase inhibitors (PARPi) has been reported to amplify inflammasome signaling through activation of a STING1 (stimulator of interferon genes 1)-dependent interferon pathway^19,20^.

Given the immunogenic nature of TNBC and the emerging evidence linking inflammasome signaling to tumor immunity and therapeutic responses, a better understanding of the prognostic relevance of inflammasome components in this subtype is warranted. In this study, we investigated the expression patterns of key inflammasome-related proteins and assessed their association with clinicopathological features and survival outcomes in a cohort of TNBC patients stratified by BRCA1 germline mutation status.

## 2. Materials and methods

### Study design and patients

This retrospective study included a total of 88 female patients diagnosed with TNBC who were treated at Barretos Cancer Hospital between 2003 and 2017 and at A.C. Camargo Cancer Center between 2000 and 2014.

The inclusion criteria were female patients with unilateral invasive breast carcinoma, diagnosis of TNBC defined as estrogen receptor (ER) expression below 1 percent, progesterone receptor (PR) expression below 1 percent, and negative human epidermal growth factor receptor 2 (HER2) status as determined by immunohistochemistry or absence of HER2 gene amplification by fluorescence in situ hybridization. Only patients without evidence of distant metastasis at diagnosis and with available formalin fixed paraffin embedded (FFPE) tumor tissue suitable for immunohistochemical analysis were included.

The study was approved by the Research Ethics Committee of A.C. Camargo Cancer Center (protocol number 2.758.540) and Barretos Cancer Hospital (protocol number 4.617.383) and was conducted in accordance with the Declaration of Helsinki. Due to the retrospective nature of the study, the requirement for informed consent was waived by the institutional review boards.

### Immunohistochemistry and tissue microarray construction

Formalin fixed paraffin embedded tumor blocks were sectioned at 5 µm and stained with hematoxylin and eosin for histopathological evaluation. All slides were reviewed by an experienced breast pathologist to confirm diagnosis and identify representative tumor areas. Manual tissue microdissection was performed to ensure that analyzed samples contained at least 70 percent tumor tissue.

Tissue microarrays were constructed using the Galileo Tissue MicroArrayer CK 4500 system. Each tumor was represented in triplicate cores to account for intratumoral heterogeneity. Consecutive TMA sections of 4 µm were subjected to immunohistochemical staining using an automated BenchMark XT platform.

Deparaffinization was performed using EZ PREP solution, followed by antigen retrieval with Cell Conditioning 1 solution at 95 degrees Celsius for 32 min for NLRP3, and Cell Conditioning 2 solution at 95 degrees Celsius for 32 min for PYCARD. Slides were incubated with primary antibodies against NLRP3, PYCARD, CASP1, and IL 18 at 37 degrees Celsius for one hour. Detection was carried out using the OptiView DAB IHC Detection Kit combined with the OptiView Amplification Kit.

After immunostaining, slides were counterstained with hematoxylin and bluing reagent, dehydrated, and coverslipped. Appropriate positive and negative controls were included in each staining run. All antibodies were validated during the pre analytical phase according to manufacturer recommendations.

### Digital image analysis and quantification

Immunohistochemical staining was quantified using digital image analysis combined with deep learning algorithms. Tumor regions were segmented, and individual cell populations were identified based on morphological features, including nuclear size, shape, and nuclear to cytoplasmic ratio. Tumor cells, lymphocytes, and stromal cells were automatically detected, and immunoreactivity was quantified according to staining intensity thresholds. Protein expression levels were calculated as the proportion of positive cells within tumor regions of interest.

### Statistical analysis

All statistical analyses were performed using R software version 4.0. Categorical variables were compared using Fisher’s exact test or the chi squared test, as appropriate. Correlations between biomarker expression levels were assessed using Spearman’s correlation coefficient.

Optimal cut-off values for each immunohistochemical marker were determined using the Evaluate Cutpoints application^21^, which identifies thresholds that best discriminate survival outcomes. Based on these thresholds, protein expression was categorized as low or high in an exploratory manner.

Disease free survival was defined as the interval between the date of surgery and the first documented recurrence or disease progression. Overall survival was defined as the time from surgery to death from any cause or last follow-up.

Univariable and multivariable Cox proportional hazards regression models were used to estimate hazard ratios and 95 percent confidence intervals. Variables included in multivariable models were selected based on clinical relevance and statistical significance in univariable analyses. The proportional hazards assumption was assessed using Schoenfeld residuals. A two sided p value below 0.05 was considered statistically significant.

## Results

### Patient clinicopathological characteristics

A total of 88 patients diagnosed with TNBC were included in this study. The predominant histological subtype was invasive ductal carcinoma (IDC), observed in 66 patients (75%). Based on BRCA1 status, 58 patients (65.8%) were classified as BRCA1 wild-type, 14 (15.9%) harbored pathogenic germline BRCA1 variants, 12 (13.6%) exhibited BRCA1 promoter hypermethylation, and 4 (4.5%) presented variants of uncertain significance (VUS).

Patients were stratified according to BRCA1 status into BRCA1-altered tumors, defined as tumors harboring pathogenic germline BRCA1 variants or BRCA1 promoter hypermethylation (n = 26), and BRCA1 wild-type/VUS tumors (n = 62). Among patients with BRCA1-altered tumors, most were aged between 41 and 69 years (15/26, 57.7%) (Table 1).

**Table 1:** Clinicopathological characteristics of patients with triple-negative breast cancer (TNBC) stratified according to BRCA1 status as BRCA1 wild-type/variants of uncertain significance (VUS) (n = 62) and BRCA1-altered tumors, defined as tumors harboring pathogenic germline BRCA1 variants and/or BRCA1 promoter hypermethylation (n = 26).

Characteristics	BRCA1 wild-type/VUS	BRCA1 methylation + gBRCA1
	(n = 62)	(n = 26)
Age (years)		
≤ 40	11 (17.7%)	9 (34.6%)
41–69	44 (71.0%)	15 (57.7%)
≥ 70	7 (11.3%)	2 (7.7%)
P value		0.22
Body mass index (kg/m^2^)		
18.5–25	32 (51.6%)	8 (30.8%)
25–30	21 (33.9%)	12 (46.2%)
> 30	9 (14.5%)	6 (23.1%)
p value		0.19
Histological type		
IDC	45 (72.6%)	21 (80.8%)
ILC	3 (4.8%)	1 (3.8%)
Other	14 (22.6%)	4 (15.4%)
P value		0.82
Histological grade		
I–II	11 (17.7%)	2 (7.7%)
III	51 (82.3%)	24 (92.3%)
P value		0.33
Axillary lymph node		
Negative	26 (41.9%)	17 (65.4%)
Positive	36 (58.1%)	9 (34.6%)
P value		0.04
Tumor size		
≤ 2 cm	15 (24.2%)	8 (30.8%)
2–5 cm	36 (58.1%)	12 (46.2%)
> 5 cm	11 (17.7%)	6 (23.1%)
P value		0.59
Family history of breast/ovarian cancer		
Negative	55 (88.7%)	15 (57.7%)
Positive	7 (11.3%)	11 (42.3%)
P value		0.001
Recurrence		
Negative	43 (69.4%)	19 (73.1%)
Positive	19 (30.6%)	7 (26.9%)
P value		0.73
NLRP3 expression		
Low	32 (52.5%)	7 (26.9%)
High	29 (47.5%)	19 (73.1%)
P value		0.03
PYCARD expression		
Low	21 (34.4%)	7 (26.9%)
High	40 (65.6%)	19 (73.1%)
P value		0.49
IL-18 expression		
Low	25 (41.0%)	5 (19.2%)
High	36 (59.0%)	21 (80.8%)
P value		0.05
CASP1 expression		
Low	26 (42.6%)	11 (42.3%)
High	35 (57.4%)	15 (57.7%)
P value		0.98

Abbreviations: BMI, body mass index; BRCA1, breast cancer susceptibility gene 1; CASP1, caspase-1; gBRCA1, germline BRCA1 pathogenic variant; IDC, invasive ductal carcinoma; ILC, invasive lobular carcinoma; IL-18, interleukin-18; NLRP3, nucleotide-binding oligomerization domain-like receptor protein 3; PYCARD, PYD and CARD domain containing; TNBC, triple-negative breast cancer; VUS, variant of uncertain significance.

Analysis of inflammasome-related protein expression revealed significantly higher levels of IL-18 (p = 0.05) and NLRP3 (p = 0.03) in tumors harboring BRCA1 alterations (pathogenic germline variants and/or promoter hypermethylation) compared with wild-type and VUS cases (Table 1).

Regarding treatment, 33 patients (37.5%) received neoadjuvant (preoperative) chemotherapy, while 27 patients (30.6%) underwent adjuvant (postoperative) chemotherapy. Treatment information was unavailable for 28 patients (31.8%).

## Expression of proteins of the inflammasome

In this study, deep learning–based image analysis tools, as detailed in the Methods section, were applied to immunohistochemistry (IHC) images to assist in the identification and quantification of distinct cellular compartments within tumor samples. Tumor cell membranes were highlighted in yellow, orange, or red according to predefined IHC intensity thresholds. Lymphocytes were identified based on nuclear size and nuclear-to-cytoplasmic ratio and are shown in pink, whereas intratumoral fibroblasts were identified by nuclear size and morphology, with nuclei highlighted in green.

**Fig. 1:**
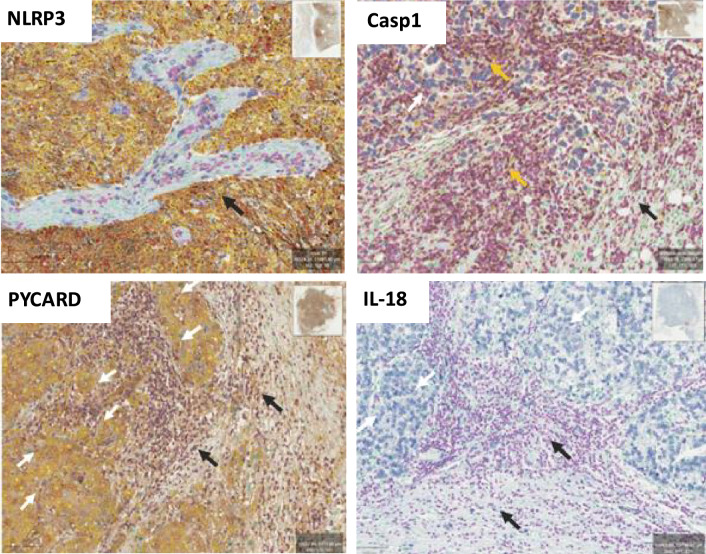
Representative immunohistochemical staining of TNBC tissue sections showing the expression of inflammasome components. Staining was performed using DAB chromogen with hematoxylin nuclear counterstaining. Tumor cell staining is indicated by white arrows, highlighting positive expression within malignant epithelial cells. Yellow arrows indicate immune cells within the tumor microenvironment, whereas black arrows highlight stromal and fibroblast-rich regions. Deep learning–based image analysis tools were used to assist in the identification of cellular compartments and IHC signal intensity, as described in the Methods. Tumor regions are shown at 400 × magnification. Scale bar = 10 µm.

As illustrated in representative images shown in Fig. 1, distinct expression patterns of inflammasome components were observed in TNBC tissues. NLRP3 exhibited predominantly membranous staining in tumor cells, whereas PYCARD was mainly localized to the tumor cell cytoplasm. CASP1 expression was primarily detected in immune cells within the tumor microenvironment. IL-18 staining was not detected in tumor cells above the predefined IHC detection threshold under the conditions used.

## Association between expression of inflammasome markers and clinicopathological parameters

A statistically significant association was observed between lower CASP1 expression and smaller tumor size (*p* = 0.005). In addition, higher NLRP3 expression was significantly associated with the presence of axillary lymph node metastasis (*p* = 0.003). A borderline association between NLRP3 expression and tumor size was also detected (*p* = 0.06) (Table 2).

**Table 2:** Association between the expression levels of inflammasome proteins (NLRP3, PYCARD, CASP1, and IL-18) and clinicopathological characteristics of patients with TNBC.

Variables	NLRP3 Low	NLRP3 High	CASP1 Low	CASP1 High	IL-18 Low	IL-18 High	PYCARD Low	PYCARD High
Age (years)								
≤ 40	6	12	13	5	5	10	11	3
40–50	21	29	40	9	15	22	30	5
> 50	3	6	6	1	3	5	6	1
P value	0.78		0.66		0.93		0.86	
Body mass index (kg/m^2^)								
18.5–25	13	25	24	5	8	20	23	4
25–30	13	15	24	8	10	13	19	3
> 30	4	7	11	2	5	4	5	2
P value	0.62		0.74		0.30		0.69	
Family history								
Negative	25	38	46	14	19	30	40	5
Positive	5	9	13	1	4	7	7	4
P value	0.78		0.27		1.00		0.06	
Tumor size								
≤ 2 cm	4	18	17	3	5	11	14	2
2–5 cm	20	22	34	4	14	20	26	5
> 5 cm	6	7	8	8	4	6	7	2
P value	0.06		0.005		0.82		0.78	
Histological type								
IDC	24	32	42	14	16	27	35	6
ILC	1	3	3	1	0	2	2	0
Other	0	6	5	0	2	3	2	1
P value	0.09		0.68		0.83		0.58	
Histological grade								
I–II	4	7	8	4	2	6	8	1
III	26	40	51	11	21	31	39	8
P value	1.00		0.25		0.70		1.00	
Axillary lymph node								
Negative	8	29	31	6	10	19	21	5
Positive	22	17	28	9	13	18	26	4
P value	0.003		0.39		0.55		0.72	
Pathogenic germline BRCA1 variant								
Absent	28	39	48	14	20	33	43	6
Present	2	8	11	1	3	4	4	3
P value	0.30		0.44		1.00		0.07	

Abbreviations: BMI, body mass index; BRCA1, breast cancer susceptibility gene 1; CASP1, caspase-1; IDC, invasive ductal carcinoma; ILC, invasive lobular carcinoma; IL-18, interleukin-18; NLRP3, nucleotide-binding oligomerization domain-like receptor protein 3; PYCARD, PYD and CARD domain containing; TNBC, triple-negative breast cancer. P values were calculated using the chi-square test or Fisher’s exact test, as appropriate.

No statistically significant associations were observed between the expression levels of inflammasome markers and other clinicopathological parameters, including age, body mass index, histological type, histological grade, and recurrence status (Table 2).

PYCARD expression showed a borderline association with pathogenic germline BRCA1 variant status (P = 0.07). However, no statistically significant associations were detected between the expression of inflammasome-related proteins and BRCA1 mutation or promoter hypermethylation status (Supplementary Table 1). Spearman correlation analysis revealed a moderate positive correlation between CASP1 and PYCARD expression (ρ = 0.37, p = 0.01), as well as a moderate inverse correlation between IL-18 and PYCARD (ρ =  − 0.41, p = 0.003). No significant correlations were observed between NLRP3 and the other inflammasome components (Supplementary Table 1).

## Association between expression of inflammasome markers and survival

**Fig. 2:**
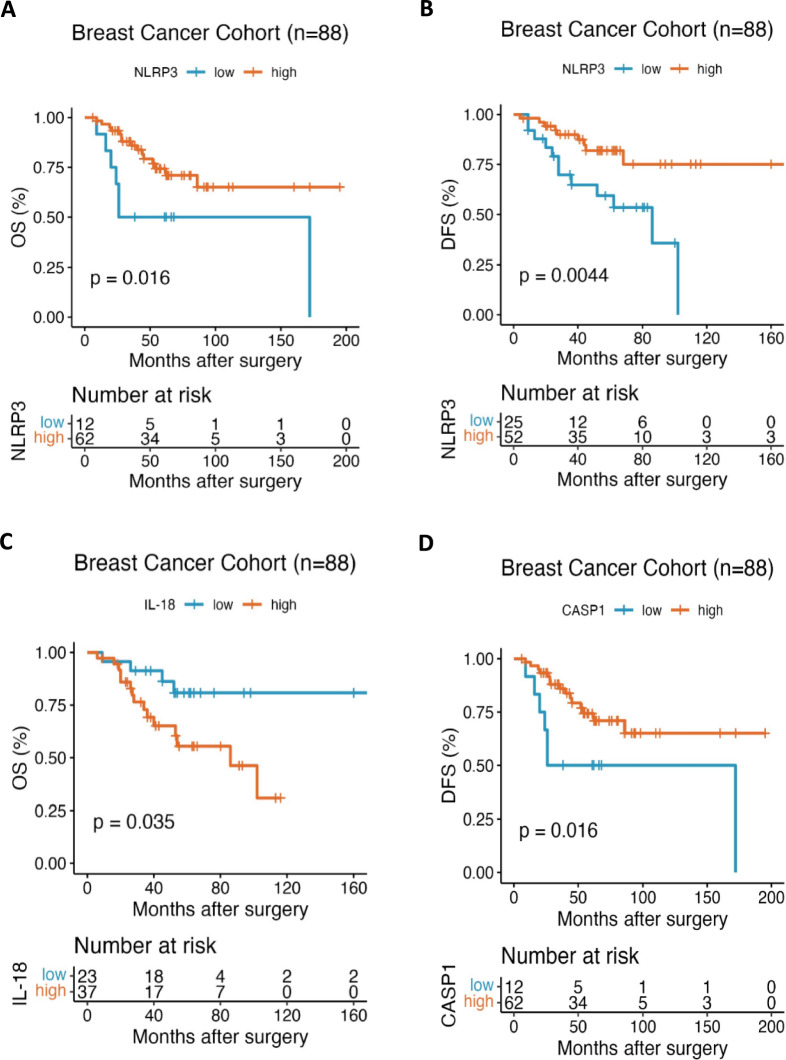
Association between inflammasome marker expression and survival outcomes in TNBC. Kaplan–Meier survival curves showing overall survival (OS) and disease-free survival (DFS) in TNBC cohort (cases with available data), stratified according to inflammasome protein expression levels. (**A**) Overall survival according to NLRP3 expression (high vs. low). (**B**) Disease-free survival according to NLRP3 expression. (**C**) Overall survival according to IL-18 expression (high vs. low). (**D**) Disease-free survival according to CASP1 expression (high vs. low). Survival differences between groups were assessed using the log-rank test. Tick marks indicate censored observations. The number of patients at risk at each time point is shown below each panel. Sample size varies across panels according to marker expression and data availability; ‘n’ represents the number of cases with evaluable immunohistochemical expression and follow-up data for each marker.

Among cases with available marker expression and follow-up data, Kaplan–Meier survival analyses were performed to evaluate the association between inflammasome marker expression and clinical outcomes. High NLRP3 expression was significantly associated with improved overall survival (OS) and disease-free survival (DFS) in patients with TNBC. Kaplan–Meier analysis demonstrated that patients with high NLRP3 expression exhibited longer OS compared with those with low expression (log-rank *p* = 0.016; Fig. 2A). Similarly, high NLRP3 expression was associated with significantly improved DFS (log-rank *p* = 0.0044; Fig. 2B).

CASP1 expression was also associated with favorable survival outcomes. While no statistically significant association was observed between CASP1 expression and OS, patients with high CASP1 expression showed significantly prolonged DFS compared with those with low expression (log-rank *p* = 0.016; Fig. 2D).

In contrast, higher IL-18 expression was associated with worse overall survival in univariate Kaplan–Meier analysis (log-rank *p* = 0.035; Fig. 2C). However, this association was not maintained in multivariable analysis, indicating that IL-18 expression was not an independent prognostic factor in this cohort.

Collectively, these findings indicate that NLRP3 expression is consistently associated with improved survival outcomes in TNBC, while CASP1 expression is significantly associated with disease-free survival. The prognostic relevance of IL-18 appears to be context-dependent and limited to univariate analysis.

## Prognostic relevance of inflammasome proteins expression in TNBC patients

Univariable Cox proportional hazards regression analysis identified several clinicopathological parameters associated with adverse survival outcomes in patients with TNBC (Table 3). Tumor size greater than 5.0 cm was strongly associated with poorer overall survival (OS) (hazard ratio [HR]: 9.9; 95% confidence interval [CI]: 3.16–30.9; *p* < 0.001) and shorter disease-free survival (DFS) (HR: 12.0; 95% CI: 3.31–43.3; *p* = 0.0002). Positive axillary lymph node status was also associated with reduced OS (HR: 2.7; 95% CI: 1.2–6.1; *p* = 0.018) and shorter DFS (HR: 3.4; 95% CI: 1.4–8.5; *p* = 0.009). In addition, a body mass index (BMI) between 25 and 30 kg/m^2^ was associated with worse OS (HR: 2.6; 95% CI: 1.1–6.0; *p* = 0.02).

**Table 3:** Univariable Cox proportional hazards regression analysis evaluating the association of clinicopathological parameters and inflammasome protein expression with disease-free survival (DFS) and overall survival (OS) in patients with TNBC.

Variables	N	Events	Median DFS (months)	HR (95% CI)	P value	N	Events	Median OS (months)	HR (95% CI)	P value
Overall	88	26	125.8	–	–	88	29	122.1	–	–
Age (years)										
≤ 40	20	6	109.2	Reference	–	20	7	104.3	Reference	–
40–50	59	18	128.6	0.83 (0.33–2.10)	0.70	59	19	127.4	0.75 (0.31–1.80)	0.52
> 50	9	2	44.9	1.02 (0.20–5.20)	0.98	9	3	41.6	1.35 (0.34–5.30)	0.67
Histological type										
IDC	66	22	123.2	Reference	–	66	25	117.8	Reference	–
ILC	4	1	56.7	0.79 (0.11–5.90)	0.82	4	1	56.7	0.65 (0.09–4.90)	0.68
Other	7	2	69.6	1.00 (0.23–4.30)	0.99	7	1	80.5	0.43 (0.06–3.20)	0.41
Tumor size (cm)										
≤ 2	23	3	154.9	Reference	–	23	4	148.3	Reference	–
2–5	48	11	116.3	2.10 (0.57–7.40)	0.27	48	12	115.0	1.70 (0.55–5.30)	0.36
≥ 5	17	12	51.8	12.0 (3.31–43.3)	< 0.001	17	13	47.1	9.90 (3.16–30.9)	< 0.001
Axillary lymph node										
Negative	43	7	152.5	Reference	–	43	9	143.9	Reference	–
Positive	45	19	92.3	3.40 (1.40–8.50)	0.009	45	20	91.4	2.70 (1.20–6.10)	0.018
Body mass index (kg/m^2^)										
18.5–25	40	9	128.9	Reference	–	40	9	128.6	Reference	–
25–30	33	12	124.6	2.10 (0.87–5.00)	0.10	33	15	109.8	2.60 (1.10–6.00)	0.02
≥ 30	15	5	69.7	1.90 (0.62–5.70)	0.26	15	5	68.7	1.80 (0.60–5.50)	0.29
Histological grade										
I–II	13	7	68.6	Reference	–	13	6	72.6	Reference	–
III	75	19	135.4	0.50 (0.21–1.20)	0.12	75	23	126.8	0.74 (0.30–1.80)	0.50
BRCA1 mutation										
Absent	74	22	108.8	Reference	–	74	26	103.9	Reference	–
Present	14	4	146.8	0.57 (0.19–1.70)	0.32	14	3	159.6	0.36 (0.11–1.20)	0.11
NLRP3 expression										
Low	25	12	24.4	3.15 (1.36–7.30)	0.007	25	12	58.7	2.63 (1.19–5.79)	0.01
High	51	10	52.7	Reference	–	51	16	43.8	Reference	–
CASP1 expression										
Low	12	7	30.8	3.09 (1.26–7.61)	0.014	12	5	53.3	7.21 (0.96–53.95)	0.05
High	61	15	45.1	Reference	–	61	24	68.1	Reference	–
IL-18 expression										
Low	48	15	36.9	5.00 (0.66–37.92)	0.12	24	8	45.7	0.39 (0.14–1.06)	0.06
High	13	1	51.2	Reference	–	37	13	48.3	Reference	–
PYCARD expression										
Low	25	4	52.7	0.35 (0.11–1.08)	0.067	6	4	39.0	Not estimable	0.99
High	31	12	32.1	Reference	–	50	15	53.8	Reference	–

Abbreviations: BMI, body mass index; CI, confidence interval; DFS, disease-free survival; HR, hazard ratio; IDC, invasive ductal carcinoma; ILC, invasive lobular carcinoma; OS, overall survival; TNBC, triple-negative breast cancer.

Regarding inflammasome-related proteins, low NLRP3 expression was significantly associated with reduced DFS (HR: 3.15; 95% CI: 1.36–7.30; *p* = 0.007) and poorer OS (HR: 2.63; 95% CI: 1.19–5.79; *p* = 0.01). Low CASP1 expression was also associated with shorter DFS (HR: 3.09; 95% CI: 1.26–7.61; *p* = 0.014), although its association with OS did not reach statistical significance and was characterized by wide confidence intervals, reflecting limited statistical power in univariable analysis. No statistically significant associations were observed between IL-18 or PYCARD expression and survival outcomes.

**Fig. 3:**
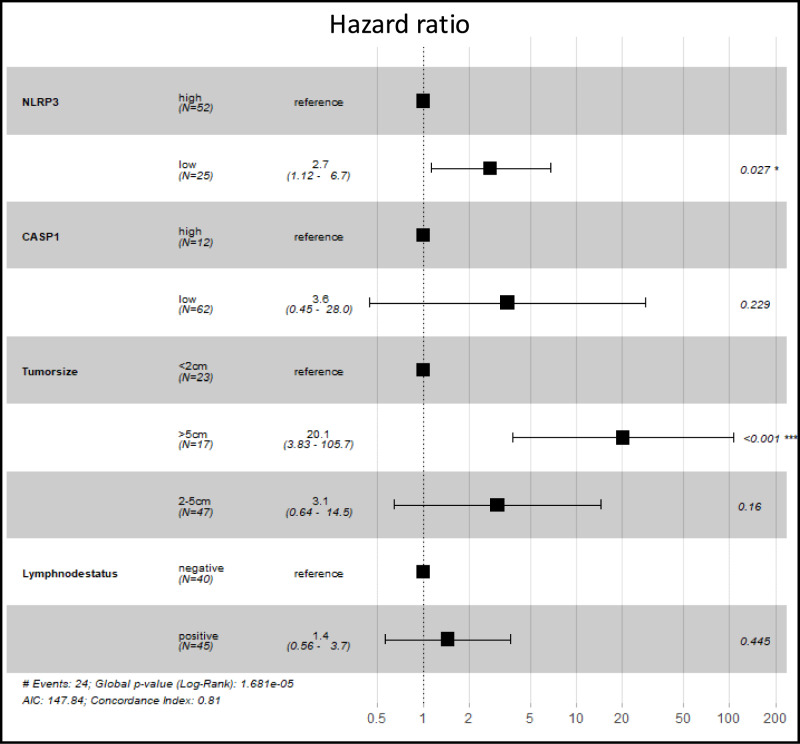
Forest plot showing hazard ratios (HRs) and 95% confidence intervals (CI) for overall survival (OS) derived from multivariable Cox proportional hazards regression analysis in patients with TNBC. Reference categories are indicated. Boxes represent HR point estimates, and horizontal lines represent 95% CI.

Multivariable Cox proportional hazards regression analysis confirmed that low NLRP3 expression (HR: 2.7; 95% CI: 1.12–6.7; *p* = 0.027) and tumor size greater than 5.0 cm (HR: 20.1; 95% CI: 3.83–105.7; *p* < 0.001) were independently associated with poorer OS (Fig. 3). By contrast, CASP1 expression and lymph node status were not independently associated with OS after multivariable adjustment. Furthermore, reduced DFS was independently associated with low NLRP3 expression (HR: 5.8; 95% CI: 1.95–17.2; *p* = 0.002), low CASP1 expression (HR: 5.3; 95% CI: 1.75–16.4; *p* = 0.003), and tumor size greater than 5.0 cm (HR: 28.6; 95% CI: 4.32–189.7; *p* < 0.001).

Given the limited sample size and the number of events in some biomarker subgroups, these findings should be interpreted cautiously and are considered exploratory. Nevertheless, the consistent association of NLRP3 expression with both DFS and OS supports its potential prognostic relevance in TNBC.

## Discussion

TNBC is characterized by marked genomic instability, pronounced immune infiltration, and a higher prevalence of BRCA1-related alterations, making it a biologically relevant context in which to investigate inflammasome-related signaling and its prognostic implications. As key sensors of innate immunity, the NLRP3 inflammasome plays a dichotomous and context-dependent role in tumorigenesis^23^. Upon activation by diverse stimuli, the inflammasome recruits the adaptor protein PYCARD and the cysteine protease CASP1^24^. Activated CASP1 promotes the maturation of pro-IL-1β and pro-IL-18 and induces gasdermin D cleavage, leading to pyroptosis, a lytic form of programmed cell death associated with the release of inflammatory mediators^12,25–27^. In the tumor microenvironment of TNBC, inflammasome-dependent cytokines such as IL-1β and IL-18 may exert both immunostimulatory and immunosuppressive effects, depending on cellular context and disease stage^28–30^.

Global expression profiling studies have shown that components of inflammasome-related signaling pathways are frequently dysregulated in breast cancer^31^. In the present study, higher NLRP3 expression was associated with axillary lymph node metastasis, suggesting a potential role for inflammasome signaling in tumor dissemination^32^. Despite this association, survival analyses consistently demonstrated that low NLRP3 expression was associated with poorer disease-free survival and overall survival. Moreover, multivariable Cox regression confirmed low NLRP3 expression as an independent adverse prognostic factor for both outcomes. Together, these findings indicate that NLRP3 expression may reflect biologically distinct inflammatory states within TNBC, with preserved NLRP3 expression potentially associated with more effective antitumor immune surveillance.

In this cohort, some patients received neoadjuvant chemotherapy, others received adjuvant chemotherapy, and treatment information was unavailable for a subset of cases. Experimental evidence suggests that certain chemotherapeutic agents can enhance tumor immunogenicity by activating inflammasome pathways and promoting the release of interleukin-1β^33^. However, the incomplete treatment data in this retrospective cohort limit definitive conclusions regarding treatment-inflammasome interactions and underscore the need for validation in prospectively controlled clinical studies.

CASP1 is a key effector of inflammasome activation and pyroptotic signaling. In this cohort, higher CASP1 expression was associated with improved disease-free survival, although it did not retain independent prognostic value for overall survival after multivariable adjustment. CASP1 expression was also associated with tumor size, supporting a link between inflammasome effector activation and tumor biology. Previous studies have reported heterogeneous roles for CASP1 across cancer types, including associations with metastatic behavior in TNBC^34^ and protective functions in colorectal cancer^35^, reinforcing the context-dependent role of inflammasome effector molecules.

Interleukin-18, an inflammasome-dependent cytokine with pleiotropic immune functions, showed an association with overall survival only in univariate analysis and did not emerge as an independent prognostic marker. This observation aligns with prior evidence demonstrating that elevated interleukin-18 levels may exert divergent effects on antitumor immunity and responses to immune checkpoint blockade, depending on tumor type and immune contexture^36,37^.

The assembly of the NLRP3 inflammasome complex requires recruitment of the adaptor protein ASC (PYCARD). Although PYCARD expression has been reported to be epigenetically downregulated in several malignancies^38^, its regulation in breast cancer remains incompletely understood. In this study, PYCARD expression exhibited borderline associations with pathogenic germline BRCA1 variants and with family history of breast or ovarian cancer, but was not associated with survival outcomes, suggesting a limited prognostic role in this cohort.

Emerging evidence supports an interaction between BRCA1 deficiency, inflammasome signaling, and innate immune pathways, including activation of the cGAS–STING axis^39–41^. These processes may contribute to homologous recombination deficiency and influence sensitivity to DNA-damaging agents and PARP inhibitors, even in BRCA-proficient tumors^19,42^.

Several limitations should be acknowledged. The retrospective design and limited sample size, particularly within BRCA1-altered subgroups, restrict statistical power and preclude definitive causal inference. In addition, incomplete treatment information and reliance on immunohistochemical protein expression without functional validation limit mechanistic interpretation. Therefore, these findings should be considered exploratory and warrant confirmation in independent, prospectively characterized cohorts of TNBC patients.

## Conclusion

In conclusion, reduced NLRP3 expression was associated with unfavorable prognosis in patients with TNBC, including shorter disease-free and overall survival. These findings support NLRP3 as a potential prognostic biomarker in this aggressive breast cancer subtype and underscore the relevance of innate immune and inflammasome-related signaling in disease progression. Although exploratory, the observed associations between inflammasome components and clinical outcomes highlight biologically distinct inflammatory states within TNBC. Further studies in independent cohorts are warranted to validate these results and to clarify the mechanistic interplay between inflammasome signaling, BRCA1 alterations, and therapeutic responses.

## Supplementary Information

Supplementary Information.

## Data Availability

The datasets used and/or analyzed during the current study are available from the corresponding author upon reasonable request.

## Abbreviations

Anti-PD-1: Anti-programmed death-1
BMI: Body mass index
CASP1: Caspase-1
DFS: Disease-free survival
DLA: Deep learning algorithms
DNMTi: DNA methyltransferase inhibitors
FFPE: Formalin-fixed and paraffin-embedded
GSDMD: Gasdermin D
H&E: Hematoxylin and eosin
HER2: Human epidermal growth factor receptor 2
HR: Hazard ratio
IDC: Invasive ductal carcinoma
IHC: Immunohistochemistry
IL-18: Interleukin-18
ILC: Invasive lobular carcinoma
MDSCs: Myeloid-derived suppressor cells
NLRs: NOD-like receptors
NLRP3: Nucleotide-binding oligomerization domain, leucine-rich repeat and pyrin domain-containing protein 3
NOD: Nucleotide-binding oligomerization domain
OS: Overall survival
PARPi: Poly(ADP-ribose) polymerase inhibitors
PD-1: Programmed death-1
PRRs: Pattern recognition receptors
PYCARD: PYD and CARD domain-containing protein
STING: Stimulator of interferon genes 1
TILs: Tumor-infiltrating lymphocytes
TME: Tumor microenvironment
TNBC: Triple-negative breast cancer
