# Supplementary material for: Analysis of the NLRP3 inflammasome components expression in triple-negative breast cancer patients with and without BRCA1 mutations
Source: Sci Rep. 2026 Mar 21;16:15316. doi: 10.1038/s41598-026-43392-0 (PMC13181052; doi:10.1038/s41598-026-43392-0)
Supplement: Supplementary file 1 — Supplementary Information. [file 41598_2026_43392_MOESM1_ESM.docx]

**Supplementary Table 1. Spearman correlation between inflammasome protein expression levels in TNBC tumors.**

|  | **NLRP3** | **CASP1** | **IL-18** | **PYCARD** |
| --- | --- | --- | --- | --- |
| **NLRP3** | 1.00 | 0.17 (p = 0.17) | −0.02 (p = 0.88) | −0.13 (p = 0.36) |
| **CASP1** | 0.17 (p = 0.17) | 1.00 | 0.08 (p = 0.53) | 0.37 (p = 0.01) |
| **IL-18** | −0.02 (p = 0.88) | 0.08 (p = 0.53) | 1.00 | −0.41 (p = 0.003) |
| **PYCARD** | −0.13 (p = 0.36) | 0.37 (p = 0.01) | −0.41 (p = 0.003) | 1.00 |

**Abbreviations:** CASP1, caspase-1; IL-18, interleukin-18; NLRP3, nucleotide-binding oligomerization domain-like receptor protein 3; PYCARD, PYD and CARD domain containing.
